# Assessing the Relationship between Hospital Process Digitalization and Hospital Quality – Evidence from Germany

**DOI:** 10.1007/s10916-024-02101-y

**Published:** 2024-09-13

**Authors:** Justus Vogel, Alexander Haering, David Kuklinski, Alexander Geissler

**Affiliations:** 1 Chair of Health Economics, Policy, and Management, School of Medicine, University of St. Gallen, St.-Jakob-Strasse 21, CH-9000 St. Gallen, Switzerland; 2 RWI – Leibniz-Institut für Wirtschaftsforschung e.V., Hohenzollernstr. 1-3, 45128 Essen, Germany

**Keywords:** Digitalization, Quality of care, Digitalization-quality relationship, Hospital process digitalization

## Abstract

Hospital digitalization aims to increase efficiency, reduce costs, and/ or improve quality of care. To assess a digitalization-quality relationship, we investigate the association between process digitalization and process and outcome quality. We use data from the German DigitalRadar (DR) project from 2021 and combine these data with two process (preoperative waiting time for osteosynthesis and hip replacement surgery after femur fracture, n = 516 and 574) and two outcome quality indicators (mortality ratio of patients hospitalized for outpatient-acquired pneumonia, n = 1,074; ratio of new decubitus cases, n = 1,519). For each indicator, we run a univariate and a multivariate regression. We measure process digitalization holistically by specifying three models with different explanatory variables: (1) the total DR-score (0 (not digitalized) to 100 (fully digitalized)), (2) the sum of DR-score sub-dimensions’ scores logically associated with an indicator, and (3) sub-dimensions’ separate scores. For the process quality indicators, all but one of the associations are insignificant. A greater DR-score is weakly associated with a lower mortality ratio of pneumonia patients (p < 0.10 in the multivariate regression). In contrast, higher process digitalization is significantly associated with a higher ratio of decubitus cases (p < 0.01 for models (1) and (2), p < 0.05 for two sub-dimensions in model (3)). Regarding decubitus, our finding might be due to better diagnosis, documentation, and reporting of decubitus cases due to digitalization rather than worse quality. Insignificant and inconclusive results might be due to the indicators’ inability to reflect quality variation and digitalization effects between hospitals. For future research, we recommend investigating within hospital effects with longitudinal data.

**Supplementary Information:**

The online version contains supplementary material available at 10.1007/s10916-024-02101-y.

## Introduction

Hospital digitalization is expected to increase the efficiency of health service delivery, reduce costs, and/ or enhance the quality of care. These expectations culminate in corresponding legislation [1–3], such as the Hospital Future Act in Germany initiating an investment funding program of more than €4 bn to digitalize German hospitals. Justification for such legislation should not only be based on favorable aims, however, but must be rooted in empirical evidence.

Such evidence regarding a positive digitalization-quality relationship is ambiguous, however. One approach is to investigate the effect of specific digitalization initiatives, e.g., the implementation of a clinical decision support system, for which reviews find a positive effect of digitalization on process quality and practitioner performance but inconclusive results regarding digitalization effects on outcome quality [4–7]. Regarding observational studies of electronic health record (EHR) adoption, early reviews suggest a positive digitalization-quality relationship [8], while more recent reviews report partially inconclusive results [9], or are debated [10–12]. In addition, recent large-scale retrospective observational studies have not shown a consistent positive relationship between digitalization and quality [13–15].

Following the structure-process-outcome quality triad of Donabedian [16], we hypothesize that higher process digitalization might increase process quality, potentially leading to better outcomes. To show an according digitalization-quality relationship, observational studies should meet three conditions:

1. The used digitalization indicator needs to be sensitive enough to detect differences in hospitals’ level of (process) digitalization.
2. Used quality indicators need to be sensitive enough to capture quality variation between hospitals.
3. A logical association between the used digitalization and quality indicators needs to be established.

We hypothesize further that one reason for the lack of conclusive evidence for a digitalization-quality relationship is that observational studies do not meet one or several of these conditions. Regarding the sensitivity of digitalization indicators, researchers commonly use categorical variables transformed into ordinal or dummy variables for EHR adoption [17–19]. Other studies use discrete digital maturity scores such as the Electronic Medical Record Adoption Model (EMRAM) stages from 0 (lowest digital maturity) to 7 (highest digital maturity) [15, 20]. These binary or ordinally scaled measures might not be flexible and detailed enough to accurately capture variations in hospital (process) digitalization.

With regards to quality indicators, used indicators might capture quality variation but clear logical associations are rarely well-grounded. Martin et al., for instance, conducted an observational study evaluating the impact of digitalization on (outcome) quality measured by five indicators aggregated at the hospital level [13]. Aggregation of quality indicators such as risk-adjusted 30-day mortality at hospital level makes it difficult to build logical associations between a digitalization indicator and the quality indicator, however, as various aspects of digitalization are averaged on the one hand and outcomes of very different patient groups and medical specialties are averaged on the other hand. In another study, Van Poelgeest et al. assessed the correlation between EMRAM stages and different composite quality measures [15]. These composite measures summarize different quality aspects of different patient groups impeding direct logical associations with digital maturity levels.

We add to the literature in three ways, aiming to fulfil the three conditions we defined above: First, we employ a sensitive digitalization indicator in our analyses, the DigitalRadar (DR) score, a continuous variable scaled between 0 (not digitalized) and 100 (fully digitalized) [21], and different DR-score sub-dimensions describing digitalization of specific processes. Second and third, we use two process and two outcome quality indicators showing quality variation between hospitals and measuring the quality of defined, relatively homogenous patient groups, complications and/ or concrete processes. With this approach, we aim to investigate the following two research questions in a novel way:

I. Is a higher level of process digitalization associated with better process quality?
II. Is a higher level of process digitalization associated with better outcome quality?

## Methods

We report our study according to the STROBE guideline (cf. supplementary material).

### Data

We use data from the DR-score dataset, collected as part of the DR evaluation project [21], from 2021, and data from the German External Inpatient Quality Assurance Program (esQS) from 2022 at the hospital site level. We use quality indicators from 2022 to avoid noise in the quality data due to COVID-19. We matched the two data sources using a unique hospital identifier and, where necessary, hospital address data. We use hospital characteristics commonly used in observational studies as controls, as they potentially influence both quality and digitalization (see Table 1).

**Table 1:** Overview and description of the model variables

Category	Source	Variable(s)	Year(s)	Description/ measurement
Process quality	esQS	Preoperative waiting time before primary hip replacement surgery after fracture of the femur (dependent variable I) – short: *Preop waiting hip replacement*	2022	Continuous variable between 0 and 100. Indicates a hospital’s share of cases that received hip replacement surgery later than 24 h after a fracture of the femur
		Preoperative waiting time before osteosynthesis surgery after fracture of the femur (dependent variable II) – short: *Preop waiting osteosynthesis*	2022	Continuous variable between 0 and 100. Indicates a hospital’s share of cases that received an osteosynthesis surgery later than 24 h after a fracture of the femur
Outcome quality	esQS	Risk-adjusted inpatient mortality ratio of patients hospitalized for outpatient-acquired pneumonia (dependent variable III) – short: *Mortality pneumonia*	2022	Continuous variable describing a hospital’s observed to expected ratio of inpatient deaths of patients hospitalized for outpatient-acquired pneumonia
		Risk-adjusted ratio of inpatient cases with a new bedsore/ decubitus, excluding decubitus/ ulcers of level/ category 1 (dependent variable IV) – short: *New decubitus cases*	2022	Continuous variable describing a hospital’s observed to expected ratio of cases developing a bedsore/ decubitus of level/ category 2 or higher during their hospital stay
Digital maturity	Digital-Radar	DigitalRadar-score (DR-score)	2021	Continuous variable between 0 (not digitalized) to 100 (fully digitalized). The DR-score is used for the first model specification.
		Five to seven DR-score sub-dimensions, depending on the investigated quality indicator: Documentation and diagnosis, decision support, access to information, telehealth emergency department, data management, order management, order and medication management, flexible working	2021	Continuous variables between 0 and 1 representing the hospital’s share of total points attained. For instance, a score of 0.52 for a sub-dimension means that a hospital attained 52% of the total score for this sub-dimension. For the second model, the individual points of all sub-dimensions logically associated with a quality indicator are summed. For the third model, sub-dimensions are included individually.
Control variables	Digital-Radar	Hospital size measured in number of beds (hospital bed categories)	2021	Dummy variables categorizing hospitals by their number of beds (less than 250, 250 to 500, 501 to 700, more than 700)
		Ownership	2021	Dummy variables indicating ownership (public, private for profit, private not-for-profit)
		Federal state	2021	Dummy variable indicating a hospital’s state
		Emergency level	2021	Dummy variables indicating the level of emergency services and of the emergency department
		Teaching hospital	2021	Dummy variable indicating whether a hospital is a teaching hospital training residents or not
		University hospital	2021	Dummy variable indicating whether a hospital is a university medical center or not

Annotations: A detailed description of the DR-dataset can be found elsewhere [21, 22]. For methodological explanations regarding the esQS indicators, we also refer to our references [23]. The esQS dataset can be obtained from the German Joint Federal Commission upon request (see online request form) [24]

The DR-score is structured into seven dimensions (e.g., systems and structures, clinical processes, telehealth, and organizational control & data management), each consisting of two to eight sub-dimensions and 28 sub-dimensions in total. We chose the DR-score as digitalization indicator and selected the two process and two risk-adjusted outcome quality indicators aiming to fulfil the conditions outlined in the introduction. Please refer to the supplementary material for (1) in-depth information on the structure, and content of the DR-score, underscoring why it might be more suitable for describing a digitalization-quality relationship than commonly used digitalization indicators (cf. *Data – DigitalRadar Score*), (2) a detailed account of the used quality indicators, including justification for why we selected these indicators (cf. *Data – Process and Outcome Quality Indicators*), and (3) an outline of the logical associations between the used digitalization and quality indicators (cf. *Data – Logical Associations*).

**Fig. 1:**
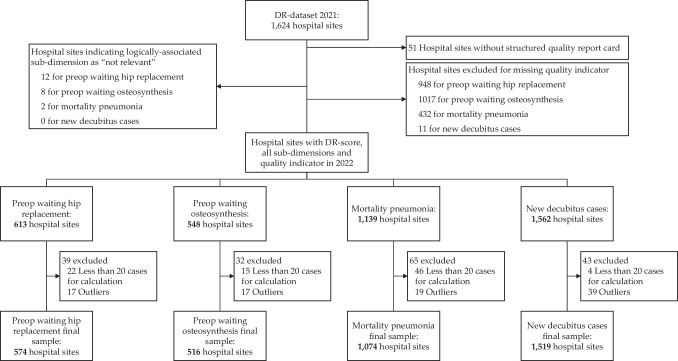
Inclusion, exclusion, and data cleaning steps to derive final samples [Annotations: For some hospitals, only one site was listed in the DR-dataset yet there was more than one site in the esQS dataset. In these cases, the same hospital’s DR-score was assigned to all sites (11 hospital sites for preop waiting hip replacement, 7 for preop waiting osteosynthesis, 49 for mortality pneumonia, and 88 for new decubitus cases)]

All data were collected at the hospital site level. The main inclusion criterion was participation in the DR-evaluation program. We excluded hospitals for which the calculation of quality indicators was based on fewer than 20 cases (cf. [23]). Lastly, hospitals were excluded if the value of their quality indicator was an outlier, i.e., if it was outside of the 95%-confidence interval of the sample median as approximated by Chambers et al. [25]. The final samples comprised 574 hospitals for preop waiting hip replacement, 516 for preop waiting osteosynthesis, 1,074 for mortality pneumonia, and 1,519 for new decubitus cases (see Fig. 1).

### Empirical Approach and Statistical Model

For each quality indicator, we provide standard descriptive statistics (e.g., arithmetic mean, standard deviation, minimum and maximum values for continuous variables) to create transparency of the dependent, explanatory, and control variables in the four samples. Besides, for each indicator, we present a correlation analysis with the total DR-score to produce first hypothetic insights into potential digitalization-quality relationships and to understand the distribution of quality indicator values and the DR-score better.

We run a univariate and a multivariate linear regression for each quality indicator. The univariate regression tests whether a quality indicator is associated with process digitalization without controls. With the multivariate linear regression, we assess whether a digitalization-quality relationship potentially found in the first regression holds after adding controls.

For a holistic consideration of hospitals’ digital maturity and process digitalization, we specify three models, changing the explanatory variable. The explanatory variables in the three models are (1) the total DR-score, (2) the sum of sub-dimensions logically associated with the respective quality indicator, and (3) the separate values of these sub-dimensions. For instance, the quality indicator preop waiting hip replacement is logically associated with five sub-dimensions (cf. *Data – Logical associations* in the supplementary material). Each sub-dimension is scaled between 0 and 1, representing the share of points achieved for that sub-dimension (cf. *Data – DigitalRadar Score* in the supplementary material). Thus, in model (2), the explanatory variable “sum of sub-dimensions” would be scaled between 0 and 5 for the indicator preop waiting hip replacement.

With this approach, we address condition (1) that differences in digitalization need to be detectable by the used digitalization indicator, as these might be more or less evident at these three measurement levels. Moreover, we address condition (3) that a strong logical association needs to be arguable between digitalization and quality indicators, as this association might be less strong for an overall digitalization measure such as the total DR-score but stronger for more detailed indicators such as the (sum of) sub-dimensions (cf. *Data – Logical associations* in the supplementary material).

All regressions can be formulated based on ordinary least squares:

$${y}_{i}={\beta }_{0}+{\beta }_{\text{d},\text{i}}{D}_{i}{\prime}+{\beta }_{\text{x},\text{i}}{X}_{i}{\prime}+{\varepsilon }_{i}$$

where $y$ is the value of the respective quality indicator of hospital site $i$. ${D}_{i}{\prime}$ is the digitalization indicator (i.e., a vector of the DR-score sub-dimensions for model (3)). ${X}_{i}{\prime}$ is a vector of control variables, only included in the multivariate regression. ${\varepsilon }_{i}$ are heteroskedasticity-robust standard errors.

We used the statistical software *R* for all calculations.

### Sensitivity Analyses

We perform two sensitivity analyses. First, to match the measurement period of the DR-score and the quality data while aiming to ensure quality indicator robustness, we run all models with the average hospital quality of 2020 and 2021. Second, we include quality indicator outliers outside of the 95%-confidence interval of the sample median in our models.

## Results

### Descriptive Results

Table 2 summarizes descriptive results.

**Table 2:** Descriptive results

	Preop waiting hip replacement (n = 574)		Preop waiting osteosynthesis (n = 516)		Mortality pneumonia (n = 1,074)		New decubitus cases (n = 1,519)	
	Mean (SD) or n (%)	Min, Max	Mean (SD) or n (%)	Min, Max	Mean (SD) or n (%)	Min, Max	Mean (SD) or n (%)	Min, Max
Respective quality indicator^1^	10.26 (5.26)	0.00, 24.19	10.90 (5.60)	0.00, 25.93	0.74 (0.43)	0.00, 1.97	0.93 (0.74)	0.00, 3.12
DR-score and sub-dimensions								
Total score^2^	10.26 (5.26)	0.00, 24.19	36.57 (9.10)	15.91, 61.24	35.12 (9.55)	7.26, 63.87	33.85 (10.25)	3.27, 63.87
Sum of sub-dimensions^3^	1.87 (0.55)	0.34, 3.54	1.89 (0.52)	0.60, 3.54	3.30 (0.88)	0.33, 5.47	1.75 (0.69)	0.03, 3.59
*Clinical processes*								
Documen./Diagnosis	0.48 (0.15)	0.04, 0.81	0.49 (0.15)	0.09, 0.81	0.46 (0.16)	0.00, 0.90	0.44 (0.17)	0.00, 0.90
Decision support	0.23 (0.18)	0.01, 0.84	0.23 (0.18)	0.01, 0.84	0.21 (0.18)	0.01, 0.69	0.20 (0.18)	0.00, 0.84
Access to information	0.70 (0.16)	0.08, 1.00	0.71 (0.15)	0.10, 1.00	0.68 (0.18)	0.00, 1.00	-	-
Order management	-	-	-	-	0.64 (0.20)	0.01, 1.00	0.59 (0.23)	0.01, 1.00
Order & med. mgt	-	-	-	-	0.21 (0.18)	0.01, 0.69	0.20 (0.17)	0.01, 0.69
Flexible working	-	-	-	-	0.78 (0.24)	0.01, 1.00	-	-
*Telehealth*								
Emergency Department	0.13 (0.16)	0.01, 0.85	0.13 (0.16)	0.00, 0.85	-	-	-	-
*Organizational control & data management*								
Data management	0.33 (0.14)	0.01, 0.73	0.33 (0.14)	0.01, 0.73	0.32 (0.14)	0.01, 0.73	0.31 (0.15)	0.01, 0.73
Hospital characteristics								
Bed category								
less than 250	163 (28%)	-	142 (28%)	-	451 (42%)	-	767 (50%)	-
250 to 500	223 (39%)	-	196 (38%)	-	344 (32%)	-	408 (27%)	-
501 to 700	87 (15%)	-	82 (16%)	-	124 (12%)	-	157 (10%)	-
more than 700	101 (18%)	-	96 (19%)	-	155 (14%)	-	187 (12%)	-
Ownership								
public	224 (39%)	-	199 (39%)	-	441 (41%)	-	551 (36%)	-
private for profit	259 (45%)	-	233 (45%)	-	422 (39%)	-	544 (36%)	-
private not-for-profit	91 (16%)	-	84 (16%)	-	211 (20%)	-	424 (28%)	-
Emergency level^4^								
no emergency level	-	-	-	-	118 (11%)		477 (31%)	
Level 1	269 (47%)	-	236 (46%)	-	512 (48%)	-	552 (36%)	-
Level 2	180 (31%)	-	166 (32%)	-	279 (26%)	-	295 (19%)	-
Level 3	125 (22%)	-	114 (22%)	-	165 (15%)	-	195 (13%)	-
Teaching hospital	444 (77%)	-	403 (78%)	-	745 (69%)	-	900 (59%)	-
University medical center	26 (4.5%)	-	24 (4.7%)	-	37 (3.4%)	-	54 (3.6%)	-
Annotations: n = number of observations. (1) Quality indicator values for preop waiting hip replacement and preop waiting osteosynthesis are continuous variables between 0 and 100. They indicate a hospital’s share of cases that received hip replacement or osteosynthesis surgery later than 24 h after a fracture of the femur. The quality indicator value for mortality pneumonia is a continuous variable describing a hospital’s observed to expected ratio of inpatient deaths of patients hospitalized for outpatient-acquired pneumonia. The quality indicator value for new decubitus cases is a continuous variable describing a hospital’s observed to expected ratio of cases developing a bedsore/ decubitus of level/ category 2 or higher during the hospital stay; (2) Continuous variable scaled between 0 (not digitalized) to 100 (fully digitalized); (3) Sub-dimensions are scaled between 0 and 1, representing the degree to which the total possible score of a sub-dimension was reached by a hospital. Thus, when summing different sub-dimensions’ scores logically associated with a quality indicator, this sum is scaled between 0 and the number of summed sub-dimensions. (4) Level 1 describes “Basic emergency care”, Level 2 “Extended emergency care”, and Level 3 “Comprehensive emergency care”[26]								

The sub-dimensions decision support, order & medication management, telehealth emergency department, and data management have relatively low sample means equal to or below 0.33 with standard deviations of roughly 50% or more of the sample means. The sub-dimensions access to information, order management, and flexible working have relatively high sample means of at least 0.59 and relatively low standard deviations. The sub-dimension documentation/ diagnosis is in between these two groups with sample means between 0.44 and 0.49 and standard deviations between 0.15 and 0.17.

**Fig. 2:**
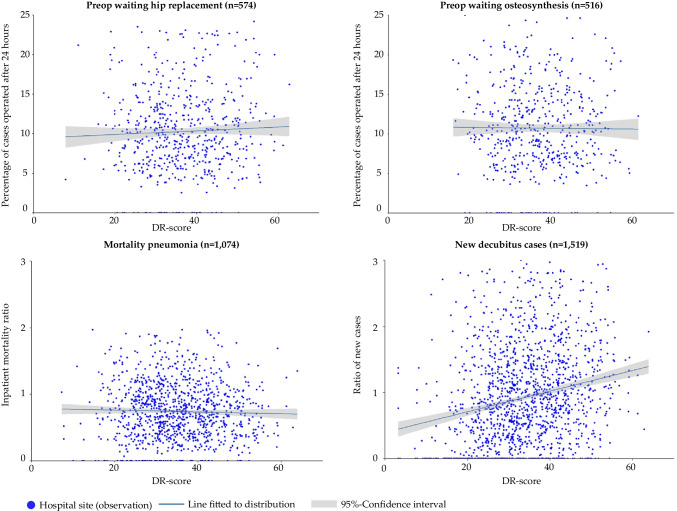
Correlation analyses of quality indicators and the DR-score [Annotations: n = number of observations. The lines in the figures display a linear fit between the x and y variables. The shaded area represents the 95% confidence interval. Each data point corresponds to a unique combination of x and y values.]

Figure 2 visualizes the correlation analyses of the quality indicators and the DR-score.

Insights from these analyses are: (1) for all four quality indicators, there are many hospitals with “perfect quality”, i.e., values equal to 0; (2) most quality indicator values are below 10 for the process and below 1.0 for the outcome quality indicators; (3) the vast majority of hospitals score between 20 and 50 DR-points; and (4) we observe no correlation between the DR-score and the quality indicators, except for the ratio of new decubitus cases which seems to be positively correlated with the DR-score.

### Statistical Model

We present model results in Table 3.

**Table 3:** Model results

	Dependent variable: Value for respective quality indicator	
	(a) Univariate regression analysis	(b) Multivariate regression analysis
Preop waiting hip replacement (n = 574)		
Model (1): Total DR-score	0.021 (0.024)	0.021 (0.027)
R^2^ (adj.)	0.000	0.040
Model (2): Sum of sub-dimensions	0.166 (0.414)	0.078 (0.459)
R^2^ (adj.)	-0.001	0.039
Model (3): Separate sub-dimensions		
*Clinical Processes*		
Documentation/ Diagnosis	-0.156 (1.940)	0.241 (2.013)
Decision support	-1.741 (1.509)	-0.963 (1.55)
Access to information	0.25 (1.613)	-0.612 (1.801)
*Telehealth*		
Emergency department	0.200 (1.359)	-0.119 (1.512)
*Organizational Control & Data Management*		
Data management	3.016* (1.827)	2.235 (1.865)
R^2^ (adj.)	-0.003	0.035
Preop waiting osteosynthesis (n = 516)		
Model (1): Total DR-score	0.001 (0.028)	-0.007 (0.031)
R^2^ (adj.)	-0.002	0.049
Model (2): Sum of sub-dimensions	-0.188 (0.466)	-0.261 (0.501)
R^2^ (adj.)	-0.002	0.049
Model (3): Separate sub-dimensions		
*Clinical Processes*		
Documentation/ Diagnosis	-0.015 (2.136)	0.062 (2.152)
Decision support	-1.583 (1.692)	-0.86 (1.705)
Access to information	-1.868 (2.047)	-1.708 (2.162)
*Telehealth*		
Emergency department	0.048 (1.505)	-0.426 (1.705)
*Organizational Control & Data Management*		
Data management	3.255 (2.148)	1.753 (2.198)
R^2^ (adj.)	0.007	0.098
Mortality pneumonia (n = 1,074)		
Model (1): Total DR-score	-0.002 (0.001)	-0.003* (0.002)
R^2^ (adj.)	0.000	0.065
Model (2): Sum of sub-dimensions	-0.005 (0.016)	-0.015 (0.016)
R^2^ (adj.)	-0.001	0.063
Model (3): Separate sub-dimensions		
*Clinical Processes*		
Documentation/ Diagnosis	-0.160 (0.131)	-0.174 (0.131)
Decision support	-0.076 (0.089)	-0.056 (0.089)
Access to information	0.288*** (0.096)	0.236** (0.096)
Order management	0.117 (0.091)	0.125 (0.091)
Order & medication management	-0.030 (0.094)	-0.081 (0.099)
Flexible working	-0.195*** (0.066)	-0.164** (0.065)
*Organizational Control & Data Management*		
Data management	0.089 (0.118)	0.069 (0.114)
R^2^ (adj.)	0.021	0.079
New decubitus cases (n = 1,519)		
Model (1): Total DR-score	0.015*** (0.002)	0.007*** (0.002)
R^2^ (adj.)	0.045	0.125
Model (2): Sum of sub-dimensions	0.218*** (0.027)	0.093*** (0.028)
R^2^ (adj.)	0.041	0.123
Model (3): Separate sub-dimensions		
*Clinical Processes*		
Documentation/ Diagnosis	0.430*** (0.163)	0.407** (0.159)
Decision support	0.337** (0.140)	0.318** (0.141)
Order management	0.320*** (0.099)	0.043 (0.099)
Order & medication management	-0.092 (0.139)	-0.261* (0.140)
*Organizational Control & Data Management*		
Data management	-0.024 (0.151)	-0.130 (0.150)
R^2^ (adj.)	0.043	0.127

Annotations: n = number of observations. In the multivariate regression analysis, we control for federal states, bed categories, ownership, emergency level, teaching hospital status and university hospital type. Coefficients are available from the corresponding author upon request. Asterisks indicate the significance level: *** p < 0.01, ** p < 0.05, * p < 0.10. Numbers in the table indicate beta coefficients, heteroskedasticity-robust standard errors are in parentheses

The two process quality indicators show insignificant associations across all model specifications (exception: data management in the univariate analysis for preop waiting hip replacement, p < 0.10).

Regarding mortality pneumonia, a higher total DR-score is weakly statistically significantly associated with a lower mortality ratio in the multivariate regression analyses (p < 0.10). Moreover, device and location independent flexible working is consistently associated with a lower mortality ratio (p < 0.01 for model (3a) and p < 0.05 for model (3b)). In contrast, access to information is associated with a higher mortality ratio for model (3a) and (3b) (p < 0.01 and p < 0.05).

The DR-score and the sum of sub-dimensions show a positive, statistically significant association with the ratio of new decubitus cases (p < 0.01 for both uni- and multivariate regression analyses). Furthermore, we observe positive, statistically significant associations with the sub-dimensions documentation/ diagnosis, decision support, and order management (only univariate regression analysis). These findings mean, however, that higher digital maturity of hospitals and relevant care processes is associated with worse outcomes which is in line with the correlation analysis (cf. Discussion). Lastly, the sub-dimension order and medication management shows a negative, weakly statistically significant association with the ratio of new decubitus cases in the multivariate regression analysis (p < 0.10).

### Sensitivity Analyses

In our first sensitivity analysis, we average the years 2020 and 2021 for the quality data to match the observation period of the digital maturity and quality data. We average two years of quality data as we suspect that the COVID-19 pandemic negatively influenced the robustness of the quality data. The main differences to our main model are:

- The total DR-score and the sum of sub-dimensions show a negative, statistically significant association with mortality pneumonia in the multivariate regression analysis (p < 0.05 and p < 0.01), while in the main model, only the total DR-score showed a weakly statistically significant association (p < 0.10).
- The sub-dimension access to information is not statistically significantly associated with mortality pneumonia in the multivariate regression analysis.
- The magnitude of the positive association of new decubitus cases and different process digitalization indicators is less strong and less significant, yet overall, the positive relationship holds.

In our second sensitivity analysis, we include quality indicator outliers outside of the 95%-confidence interval of the sample median. Main differences are that for mortality pneumonia, the total DR-score is slightly more strongly statistically significant in the multivariate analysis (p < 0.05), and the sub-dimension data management shows a positive, statistically significant relationship with mortality pneumonia (p < 0.05). Lastly, the positive relationship between process digitalization indicators and new decubitus cases is less strong and when controlling for hospital characteristics, only the total DR-score is weakly statistically significantly associated with the quality indicator (p < 0.10).

## Discussion

We test the association of hospital process digitalization measured in three different ways with two process and two outcome quality indicators. Our main model results do not show a consistent digitalization-quality relationship across indicators. Regarding the two process quality indicators, all but one of the associations were insignificant. For mortality pneumonia, we found some evidence that higher (process) digitalization is associated with better outcomes. Although statistically significant, the magnitude of this association is rather small: A one-point increase in the total DR-score, scaled between 0 and 100, is associated with a 0.004 points lower mortality ratio. For mortality pneumonia, this negative association is not consistent when considering the sub-dimensions: The association with the sum of sub-dimensions is statistically insignificant and we find both positive and negative associations with single sub-dimensions.

Regarding new decubitus cases, greater process digitalization was associated with worse quality, albeit rather weak in magnitude with regards to models (1) and (2). Still, this finding is already visible in the correlation analysis and consistent across all three model specifications. Model (3) shows that the sub-dimension documentation/ diagnosis seems to account for a large part of the positive association as it is both strong in magnitude and statistical significance. This might hint at the fact that the observed positive association could in fact not be due to actually worse quality, i.e., a higher number of unexpected new cases developing decubitus, but rather a better diagnosis, documentation, and reporting of decubitus cases. This is supported by the results of the second sensitivity analysis including quality indicator value outliers as with the inclusion of these qualitatively worst performing hospitals, statistical significance weakens strongly, and most associations become insignificant in the multivariate analysis.

We discuss our findings along conditions (2) and (3) specified in the introduction (for why we believe we comply with condition (1), i.e., digitalization indicators must be capable to measure variation in hospital (process) digitalization, see *Data – DigitalRadar Score* in the supplementary material). Regarding condition (2), namely the statistical sensitivity of the quality indicators used, our descriptive results show that there are many observations of “perfect” quality (i.e., values of 0). Moreover, observations of low quality (values greater than 15 for the process quality indicators; values greater than 1.0 and especially 2.0 for risk-adjusted ratios) are scarce. When including quality indicator outliers (i.e., very low quality) in our robustness check, our findings do not change. Therefore, we suspect that the quality indicators cannot detect quality differences between hospitals sufficiently well – at least not in the context of our research questions. The esQS program is a quality monitoring program that was developed to detect the worst hospitals in order to engage them in structured quality dialogs [23]. Moreover, all quality indicators were self-reported. These two facts might explain why we do not observe many examples of (very) low quality, affecting our ability to find statistically significant associations between process digitalization and hospital quality.

Condition (3) implies that the causal pathway between digitalizing processes and the investigated quality indicators should be explained. For transparency, we provide one example of our selection and matching logic. For instance, we argue that preoperative waiting time of hip replacement and osteosynthesis surgery patients is linked to the DR-question “In the emergency room, patient admission, triage, medical orders, and documentation tasks are carried out digitally. This is done via the [hospital information system] or special systems with interfaces to the [hospital information system]” [22]. We hypothesize that digitalization of these emergency room processes (admission, triage, etc.) might lead to faster decision-making, decreasing the preoperative waiting time for emergency and urgent surgeries, including hip replacement and osteosynthesis surgery after femur fracture which classify as urgent surgeries. Thus, we included the question’s sub-dimension (i.e., documentation/ diagnosis) in models (2) and (3).

While our assumptions regarding the digitalization-quality mechanism might be valid, we believe that a problem might arise from how process quality is measured in the esQS program. While process digitalization might decrease pre-operative waiting time measured in minutes or hours, the esQS quality indicators measure the share of cases receiving surgery later than the pre-defined threshold of 24 h after admission, which is much harder to affect with process digitalization.

### Findings from the Literature

Martin et al. analyzed the association of the NHS Clinical Digital Maturity Index (CDMI), scaled between 0 (not digitalized) to 1400 (fully digitalized), and its three dimensions, readiness, capability (most closely related to process digitalization), and infrastructure, with five different quality measures [13]. For the 30-day hospital level mortality index, the percentage of episodes of care with complications, and the number of emergency readmissions within 28-days of discharge, the authors found no significant associations. In contrast, risk-adjusted long length of stay was positively associated with the CDMI score while a higher CDMI score was associated with more harm free episodes of care. Overall, these findings are in line with our findings.

Van Poelgeest et al. examined the relationship between EMRAM stages and the “Elsevier best hospitals score”, a composite measure based on 542 quality indicators, as well as the overall Elsevier score’s three domains – medical care, patient orientation, and effective treatment [15]. The authors only find insignificant relationships which might be evidence for the importance to fulfil the conditions we defined for investigating a digitalization-quality relationship. With respect to condition (3), the authors do not provide specific hypotheses for a match of their digital maturity measure and quality indicators. Regarding condition (1), the EMRAM stages are ordinally scaled from 0 to 7, and a hospital can only advance to the next stage if it complies with all the requirements of the former stage [20]. Thus, these stages can detect major differences only in digital maturity milestones rather than processes’ digital maturity. Finally, concerning condition (2), the authors used “bundled” values for the quality measures, in the form of an ordinal scale from 1 to 4 for composite measures comprising lots of different indicators. Detecting quality variation between hospitals with such quality measures might be difficult in the context of investigating a digitalization-quality relationship.

Motivated by the above findings, we specified three models in our study changing the explanatory variable for hospital process digitalization. Martin et al. argued, for instance, that the inconsistent relationship they found might be due to the fact that the CDMI score and its dimensions did not measure all quality-relevant aspects of digitalization. We suspected the same regarding our model (1), i.e., when only considering the total DR-score. We aimed at alleviating this issue by including specific aspects of process digitalization in our models (2) and (3). As sub-dimensions are measured between 0 and 1, our models (2) and (3) might in turn have drawbacks regarding condition (1) for showing a digitalization-quality relationship, i.e., while describing process digitalization at a detailed level, they might not measure variation between hospitals sufficiently.

With longitudinal data, within hospital changes rather than between hospital differences could be assessed, e.g., with fixed effect models, addressing the challenges discussed above. Such data will become available after the second measurement period of the DR evaluation project will have been completed in the second half of 2024. Furthermore, future research could assess the relationship between process digitalization and measures commonly used in intervention studies (e.g., [27]) such as time saved in a process, reduced waiting time, or changes in utilization. However, finding nationwide data of such measures might be challenging. Thus, we argue that when developing national programs to measure hospitals’ digital maturity, suitable effectiveness measures, e.g., routinely available in hospital information systems, should be collected along with digital maturity data.

### Limitations

All esQS quality indicators were self-reported. This might create reporting bias. Similarly, the DR-score is based on a questionnaire; i.e., it is also a self-reported measure. Still, reporting bias might be rather small for the DR-score as hospitals had an incentive to answer questions truthfully [21]: After participating in the evaluation, hospitals could use a benchmarking tool allowing them to compare their own digital maturity to that of their peers. If questions were deliberately answered incorrectly, this tool would become worthless for a hospital. In addition, results were checked for plausibility and completeness, further alleviating concerns regarding reporting bias (cf. *Data – DigitalRadar Score* in the supplementary material).

We included a one year time lag in our main model. Process and outcome quality might be affected by digitalization only in the mid- to long-term, however. One reason for this is because digitalization of structures and processes needs to be accompanied by educating and training staff and process owners must implement and get accustomed to optimized, digitalized processes. Unfortunately, in the DR-dataset, we cannot identify for how long a certain structure or process had already been digitalized. New measurements and longitudinal data could also help with this issue (cf. above).

## Conclusion

Our models did not reveal a consistent digitalization–quality relationship across the four quality indicators we investigated. Regarding the two investigated process quality indicators, all but one relationship were insignificant. For mortality pneumonia, we found that a higher DR-score might be associated with better results, yet the magnitude is small. Lastly, more digitalized processes seem to be associated with more new decubitus cases, yet this relationship could also be explained by better diagnosis, documentation, and reporting of decubitus cases due to digitalization.

While these findings are in line with those of other observational studies, this result seems unsatisfactory considering the (supposed) intuitive logic of a digitalization-quality relationship, findings from intervention studies investigating the effects of specific digitalization initiatives, and last but not least the policy-driven overarching goal of healthcare digitalization.

From our findings and our discussion, we conclude that process quality indicators need to be developed and first and foremost measured in a way apt to reflect digitalization and to detect quality variation. Moreover, future research should employ longitudinal data, potentially capable of addressing shortcomings of existing studies, to find within hospital changes of quality due to digitalization, and, where possible, use effectiveness measures strongly linked to process digitalization.

## Supplementary Information

Below is the link to the electronic supplementary material.

Supplementary file1 (PDF 443 KB)

## Data Availability

The dataset from the external inpatient quality assurance program supporting this study is available upon request from the German Joint Federal Commission at Referenzdatenbank Qualitätsberichte (g-ba.de). At the time of manuscript preparation, the data request process for the DigitalRadar dataset was not finalized by the responsible institutions. Interested researchers can contact the corresponding author for more information and the current status regarding data availability.

## Code Availability

The underlying code for this study is not publicly available but may be made available to qualified researchers on reasonable request from the corresponding author.
