# Supplementary material for: Assessing the Relationship between Hospital Process Digitalization and Hospital Quality – Evidence from Germany
Source: J Med Syst. 2024 Sep 13;48(1):85. doi: 10.1007/s10916-024-02101-y (PMC11399181; doi:10.1007/s10916-024-02101-y)
Supplement: Supplementary file 1 — Supplementary file1 (PDF 443 KB) [file 10916_2024_2101_MOESM1_ESM.pdf]

Supplementary Material

Title: Assessing the relationship between hospital process digitalization hospital quality – evidence from Germany

Journal: Journal of Medical Systems

Authors: Vogel, Justus; Haering, Alexander; Kuklinski, David; Geissler, Alexander

Corresponding Author: Vogel, Justus; Chair of Health Economics, Policy and Management; School of Medicine; University of St. Gallen; St.-Jakob-Strasse 21, CH-9000 St. Gallen, Switzerland; Justus.Vogel@unisg.ch

STROBE Checklist

	Item No.	Recommendation	Page No. / Part of manuscript	Relevant text from manuscript
**Title and abstract**	1	(*a*) Indicate the study's design with a commonly used term in the title or the abstract	Abstract	For each indicator, we run a univariate and a multivariate regression.
		(*b*) Provide in the abstract an informative and balanced summary of what was done and what was found	Abstract	See "Methods" and "Results" parts in the abstract.
**Introduction**				
Background/ rationale	2	Explain the scientific background and rationale for the investigation being reported	First two paragraphs and middle section of the introduction	From "Hospital digitalization […]" to "[…] digitalization and quality." and section "[…] With regards to quality indicators, […] logical associations with quality indicators."
Objectives	3	State specific objectives, including any prespecified hypotheses	Third, fourth, and last paragraph of the introduction	Section "Following the structure-process-quality triad of […] variations in hospital (process) digitalization." and section "We add to the literature […] with better outcome quality?"
**Methods**				
Study design	4	Present key elements of study design early in the paper	Empirical Approach and Statistical Model	Entire section "Empirical Approach and Statistical Model"
Setting	5	Describe the setting, locations, and relevant dates, including periods of recruitment, exposure, follow-up, and data collection	Data	Entire section "Data"
Participants	6	*Cohort study*—Give the eligibility criteria, and the sources and methods of selection of participants. Describe methods of follow-up<br>*Case-control study*—Give the eligibility criteria, and the sources and methods of case ascertainment and control selection. Give the rationale for the choice of cases and controls<br>*Cross-sectional study*—Give the eligibility criteria, and the sources and methods of selection of participants	Data, especially Table 1 and Figure 1	For instance paragraph starting with "All data were collected at the hospital site level. The main inclusion criterion was […]"
Variables	7	Clearly define all outcomes, exposures, predictors, potential confounders, and effect modifiers. Give diagnostic criteria, if applicable	Data, Empirical Approach and Statistical Model	See Table 1 and accompanying text See paragraph starting with "For a holistic consideration of hospitals' digital maturity and process digitalization, […]".
Data sources/ measurement	8	For each variable of interest, give sources of data and details of methods of assessment (measurement). Describe comparability of assessment methods if there is more than one group	Data, Empirical Approach and Statistical Model	See Table 1 and accompanying text See paragraph starting with "For a holistic consideration of hospitals' digital maturity and process digitalization, […]".
Bias	9	Describe any efforts to address potential sources of bias	-	-
Study size	10	Explain how the study size was arrived at	Data	See Figure 1 and accompanying text
Quantitative variables	11	Explain how quantitative variables were handled in the analyses. If applicable, describe which groupings were chosen and why	Data, Empirical Approach and Statistical Model	Entire text of both sections

Statistical methods	12	(*a*) Describe all statistical methods, including those used to control for confounding	Empirical Approach and Statistical Model	Entire text of section
		(*b*) Describe any methods used to examine subgroups and interactions	Irrelevant for our study	Irrelevant for our study
		(*c*) Explain how missing data were addressed	Irrelevant for our study	Irrelevant for our study
		(*d*) *Cohort study*—If applicable, explain how loss to follow-up was addressed<br>*Case-control study*—If applicable, explain how matching of cases and controls was addressed<br>*Cross-sectional study*—If applicable, describe analytical methods taking account of sampling strategy	Irrelevant for our study	Irrelevant for our study
		(*e*) Describe any sensitivity analyses	Sensitivity analyses	Entire text of section
**Results**				
Participants	13	(a) Report numbers of individuals at each stage of study—eg numbers potentially eligible, examined for eligibility, confirmed eligible, included in the study, completing follow-up, and analysed	Irrelevant for our study	Irrelevant for our study
		(b) Give reasons for non-participation at each stage	Irrelevant for our study	Irrelevant for our study
		(c) Consider use of a flow diagram	Irrelevant for our study	Irrelevant for our study
Descriptive data	14	(a) Give characteristics of study participants (eg demographic, clinical, social) and information on exposures and potential confounders	Descriptive results	See Table 2 and accompanying text<br>See Figure 2 and accompanying text
		(b) Indicate number of participants with missing data for each variable of interest	Irrelevant for our study	Irrelevant for our study
		(c) *Cohort study*—Summarise follow-up time (eg, average and total amount)	Irrelevant for our study	Irrelevant for our study
Outcome data	15	*Cohort study*—Report numbers of outcome events or summary measures over time	Descriptive results	See Table 2 and accompanying text
		Case-control study—Report numbers in each exposure category, or summary measures of exposure		
		Cross-sectional study—Report numbers of outcome events or summary measures		
Main results	16	(*a*) Give unadjusted estimates and, if applicable, confounder-adjusted estimates and their precision (eg, 95% confidence interval). Make clear which confounders were adjusted for and why they were included	Statistical model	Entire text of section
		(*b*) Report category boundaries when continuous variables were categorized	Irrelevant for our study	Irrelevant for our study
		(*c*) If relevant, consider translating estimates of relative risk into absolute risk for a meaningful time period	Irrelevant for our study	Irrelevant for our study
Other analyses	17	Report other analyses done—eg analyses of subgroups and interactions, and sensitivity analyses	Sensitivity analyses	Entire text of section
**Discussion**				
Key results	18	Summarise key results with reference to study objectives	Discussion	First ~2 pages of the discussion
Limitations	19	Discuss limitations of the study, taking into account sources of potential bias or imprecision. Discuss both direction and magnitude of any potential bias	Discussion	Entire text of section “Limitations”
Interpretation	20	Give a cautious overall interpretation of results considering objectives, limitations, multiplicity of analyses, results from similar studies, and other relevant evidence	Discussion	Last paragraph before section “Findings from the literature” and last two paragraphs of this section

Generali-sability	21	Discuss the generalisability (external validity) of the study results	Discussion	Last two paragraphs before limitations
**Other information**				
Funding	22	Give the source of funding and the role of the funders for the present study and, if applicable, for the original study on which the present article is based	Declarations	No funding was received for conducting this study.

Data – DigitalRadar Score

The DR-score is a standardized tool measuring the digital maturity of hospitals based on a questionnaire [1, 2]. It is scaled continuously between 0 (not digitalized) and 100 (fully digitalized). The DR-score is calculated from self-reported data which hospitals could supply via an online platform. The DigitalRadar Hospital Consortium provided a support team assisting hospitals during the data collection process. Besides, the consortium conducted plausibility and quality control of answers supplied by the participating hospitals. If necessary, hospitals were contacted to correct (add) implausible (missing) information.

In the first measurement period between October and December 2021, the survey had 234 questions. Only 199 questions are relevant for the calculation of the score, however, as 35 questions ask for structural information such as the full-time equivalents of the IT department. Moreover, depending on the hospital type and infrastructure, not all 199 questions are relevant for the score. For psychiatric hospitals, for instance, questions relating to acute somatic care are irrelevant or for hospitals without emergency care department, questions relating to emergency care are irrelevant. Thus, for such questions, hospitals had the option to select "not relevant" as answer option. The DR-score of each hospital is normalized to the scale of 0 to 100 to allow for standardized comparisons while only considering questions relevant to a hospital's operations. For some questions, hospitals could select the answer option "Don't know" which was counted as 0.0 points.

The DR-score is structured into seven dimensions and each dimension is categorized into different sub-dimensions (see Table 5):

Table 5: Dimensions, and sub-dimensions of the DigitalRadar score

DR dimension	DR sub-dimensions	Number of DR sub-dimensions
Structures and systems	Software applications	1
Resilience management and performance	IT-security<br>Resilience<br>Performance and employee satisfaction	3
Organizational control and data management	Organizational control<br>Data management	2
Clinical processes	Access to information<br>Documentation and diagnosis<br>Order management<br>Quality and risk management<br>Clinical decision support<br>Flexible working<br>Order and medication management<br>Blood and sample management	8
Information exchange	Interoperability and international standardization<br>Integration of medical appliances<br>Information exchange between clinical staff<br>Information exchange with external players<br>Information exchange with patients	5
Telehealth	Emergency care unit<br>Teleconsultations<br>Telemedical networks	3
Patient participation	Strategy<br>Access to information<br>Participation possibilities<br>Usage metrics	4

Annotations: The DR dimension "structures and systems" has two more sub-dimensions "hospital information and metrics" and "IT metrics" which are not relevant for scoring.

In the DR-score dataset, dimensions and sub-dimensions are scaled between 0 and 1, representing the degree to which the total possible score of a (sub-) dimension was reached by a hospital. Thus, when summing different sub-dimensions' scores logically associated with a quality indicator, this sum is scaled between 0 and the number of summed sub-dimensions.

The total DR-score is based on the scores of the dimensions with different weights per dimension. These weights were defined by an expert advisory board including representatives from hospitals, payers, umbrella organizations of interest groups (e.g., physicians) and international digital health experts. According to the DR interim report, the expert advisory board was continuously involved in the development of the DR-score methodology [1].

In our study, we rely on the DR-score due to two advantages compared to commonly used digitalization indicators. First, the total DR-score and its (sub-) dimensions are continuously measured. Thus, it allows for nuanced variation in its measurement. Ordinal and binary measurements, such as the EMRAM stages or EHR adoption levels, follow normative criteria to reach the next level. If not all criteria of the

previous level are met, the hospital will not advance, even if some aspects of higher levels are already reached [3], limiting the measured variation and level of detail. In our view, this variation and level of detail are needed for analyzing a digitalization-quality relationship, however. Along with its (sub-) dimensions, the DR-score supplies this level of detail as well as needed variation, being almost normally distributed with the majority of hospitals in close range of the overall average (cf. the DigitalRadar Hospital Consortium interim report [1] and a publication using the DR-score for investigating a profitability-digitalization relationship [4]). Second, sub-dimensions reveal specific aspects of hospital process digitalization for which logical associations with process quality can be established (cf. section *Data – Logical associations* below).

Data – Process and Outcome Quality Indicators

Description and scale of chosen quality indicators

We use the two process quality indicators "Pre-operative waiting time before primary hip replacement surgery after fracture of the femur" (short: Preop waiting hip replacement) and "Pre-operative waiting time before osteosynthesis surgery after fracture of the femur" (short: Preop waiting osteosynthesis) for our analysis. These indicators assess the preoperative waiting time for patients with femur fractures. Minimizing preoperative waiting time for these patients is essential both for optimal pain management as well as mortality, perioperative complications, and revisions [6–8]. Thus, in the esQS program, a goal of less than 24 hours preoperative waiting time is set for hospitals. Both process indicators are continuous variables from 0 to 100. A value of 10, for instance, means that the requirement of a preoperative surgery time of less than 24 hours was not met in 10% of the cases [12, 13].

We use the two outcome quality indicators "Risk-adjusted inpatient mortality ratio of patients hospitalized for outpatient-acquired pneumonia" and "Risk-adjusted ratio of inpatient cases with a new bedsore/ decubitus, excluding decubitus/ ulcers of level/ category 1" for our analysis. The first outcome quality indicator assesses the inpatient mortality of patients that were hospitalized for outpatient-acquired pneumonia, excluding patients hospitalized with a palliative therapy goal [9]. In total, 13 risk factors significantly associated with inpatient mortality are used for risk-adjustment (e.g., age, gender,

chronic bed confinement, mean arterial blood pressure at admission, etc.). The second outcome quality indicator assesses the quality of nursing processes by measuring the ratio of observed to expected newly developed bedsores [10]. Ten risk factors significantly associated with the development of bedsores are considered for risk-adjustment (e.g., age, number of ventilation hours, obesity, diabetes, infections, etc.).

Both outcome quality indicators are ratios of the observed over the expected number of instances. Thus, a value of 1.0 implies that exactly the number of expected instances (i.e., deaths or new cases with decubitus) were also observed. Consequentially, a ratio of less than 1.0 implies better than expected quality and generally, the lower the ratio, the better the quality of a hospital.

Reasoning for inclusion of chosen quality indicators

In the German External Inpatient Quality Assurance Program (esQS), 202 quality indicators were measured and made publicly available in 2022 [5]. We selected process quality indicators according to three reasons: (1) Adequacy for testing our hypothesis regarding the influence of process digitalization on process quality and on outcome quality, (2) sufficient quality variation between hospitals, addressing condition 2 outlined in our introduction, and (3) balancing different patient groups and process areas. Moreover, the selected quality indicators needed to be suitable for building logical associations with DR-score sub-dimensions (see next section *Data – Logical Associations*).

Firstly, as outlined in the introduction, we hypothesize that process digitalization might influence process quality and ultimately outcome quality. To test this hypothesis, we include two process quality indicators strongly linked to outcome quality [6–8]. With the analysis of the two process quality indicators, we test the first part of the relationship, i.e., the direct relationship of process digitalization and process quality. We include two outcome quality indicators strongly linked to process quality [9, 10] in our analysis to test the second part of our hypothesis, i.e., the indirect relationship of process digitalization and outcome quality. We chose the quality indicator "risk-adjusted inpatient mortality ratio of patients hospitalized for outpatient-acquired pneumonia" as the esQS additionally measures six process indicators for outpatient-acquired pneumonia, underscoring the strong link between process and outcome for this indication. Regarding the indicator "risk-adjusted ratio of inpatient cases with a

new bedsore/ decubitus, excluding decubitus/ ulcers of level/ category 1”, the main lever to avoid the development of new cases is adequate decubitus prophylaxes, typically carried out by nurses. Thus, this indicator assesses the quality of a key nursing care process. Ideally, all indicators would target the same indication and/ or procedure. Still, empirical evidence for the structure-process-outcome quality triad is scarce and it is difficult to show with secondary data (e.g., [11]).

Secondly, we compared descriptive statistics of dozens of quality indicators, i.e., mean, standard deviation, median, 25th and 75th percentile, to only select indicators with relatively high variation between hospitals. The four selected indicators were among the quality indicators that showed the highest variation (cf. *Results – Descriptive Statistics* in the main paper). Still, as we outline in the Discussion part of the main paper, the process quality indicators might in fact not be apt to sufficiently unveil quality variation between hospitals.

Lastly, with our quality indicator selection we also aimed to balance surgical and non-surgical patients and (pre-) surgery, physician, and nursing focused care processes (see Table 6).

Table 6: Focused patient group(s) and care processes per quality indicator

Quality indicator	Focused patient group(s)	Focused care processes
Pre-operative waiting time before primary hip replacement surgery after fracture of the femur	Surgical patients	(Pre-) surgery processes involving physicians, nurses, and physician assistants [12]
Pre-operative waiting time before osteosynthesis surgery after fracture of the femur	Surgical patients	(Pre-) surgery processes involving physicians, nurses, and physician assistants [13]
Risk-adjusted inpatient mortality ratio of patients hospitalized for outpatient-acquired pneumonia	Non-surgical patients	Physician processes (e.g., complete realization of standard diagnostic measures) [9]
Risk-adjusted ratio of inpatient cases with a new bedsore/ decubitus (excl. decubitus/ ulcers of level/ category 1)	All patient groups	Nursing [10]

Annotations: See indicated references for more information.

We acknowledge that the disadvantage of our approach is that our findings are limited to the investigated quality indicators. Still, with our study we hope to supply a generalizable approach for investigating digitalization-quality relationships, applicable to other indicators from the esQS program and similar data collected in other countries.

Data – Logical Associations

To meet condition (3) outlined in the introduction, we linked single questions, sub-dimensions, and dimensions of the DR questionnaire [2] with the hospital processes relevant for the selected indicators, relying on similar considerations about a digitalization-quality relationship as Atasoy et al. [14]. The authors list clinical decision support, improved clinical communication, improved information management, and information exchange between providers and across sectors as the main levers for improving quality of care. These levers are part of the following DR-score dimensions and their sub-dimensions:

- Clinical processes: Documentation/ diagnosis, access to information, decision support, order management, order and medication management, device and location independent flexible working
- Telehealth: Emergency department
- Organisational control and data management: Data management

In Table 7 below we present examples of the DR questions and answer options justifying the logical association of quality indicators and the above sub-dimensions. For instance, preoperative waiting time for urgent and emergency cases admitted through the emergency care unit often accrues because medical staff in the emergency room does not have sufficient information about arriving patients. In the sub-dimension "Emergency department" of the "Telehealth" dimension, there are several questions targeting the digital information flow between ambulances, upstream providers, and the emergency care department. It is evident from the questions that the goal of this information exchange is faster diagnostic and therapeutic treatment of patients arriving in the emergency care department. Therefore, we include this sub-dimension in model specifications (2) (sum of logically associated sub-dimensions) and (3) (separate consideration of logically associated sub-dimensions).

Table 7: Logical matching of DR-sub-dimensions and quality indicators

Quality indicator	DR dimen-sion	DR sub-dimension	Examples of relevant DR question(s) and answers
Pre-operative waiting time before primary hip replacement surgery after fracture of the femur<br><br>AND<br><br>Pre-operative waiting time before osteo-synthesis surgery after fracture of the femur	Clinical processes	Documentation/ Diagnosis	In the emergency room, patient admission, triage, medical orders, and documentation tasks are carried out digitally. This is done via the KIS/KAS or special systems with interfaces to the KIS/KAS. - (1) No emergency room available, (2) Not implemented to (6) Fully implemented, (7) Don't know
		Access to information	The data exchange in the emergency room enables the uninterrupted transmission of relevant data (including NFDM, according to the interoperability directory), control of processes of emergency care (ambulance service, emergency room, emergency room) and bed management. - (1) No emergency room available, (2) Not implemented to (6) Fully implemented, (7) Don't know
			Please indicate to what extent (percentage) the following information can be accessed digitally and in a structured manner by clinical staff via the HIS in the patient context:<br>Laboratory test results - (1) 0%; (2) 1-25%; (3) 26-49%; (4) 50-75%; (5) 76-94%; (5) 95-100%; (6) Not relevant / no access required
			Please indicate to what extent (percentage) the following information can be accessed digitally and in a structured manner by clinical staff via the HIS in the patient context:<br>Radiology imaging results - (1) 0%; (2) 1-25%; (3) 26-49%; (4) 50-75%; (5) 76-94%; (5) 95-100%; (6) Not relevant / no access required
			Please indicate to what extent (percentage) the following information can be accessed digitally and in a structured manner by clinical staff via the HIS in the patient context:<br>Cardiology imaging results - (1) 0%; (2) 1-25%; (3) 26-49%; (4) 50-75%; (5) 76-94%; (5) 95-100%; (6) Not relevant / no access required
			Please indicate to what extent (percentage) the following information can be accessed digitally and in a structured manner by clinical staff via the HIS in the patient context:<br>DICOM-based images (especially from radiology and cardiology) - (1) 0%; (2) 1-25%; (3) 26-49%; (4) 50-75%; (5) 76-94%; (5) 95-100%; (6) Not relevant / no access required
			For emergencies/emergency situations, there is a defined documentation process to ensure the integrity of patient data and to be able to prove and check services or care measures provided. - (1) No emergency room available; (2) Not implemented to (6) Fully implemented; (7) Don't know
	Telehealth	Emergency department	In the hospital emergency room, digital applications are used in real time to exchange structured information or documents with emergency services, control centers and any upstream service providers. For example, information can be exchanged before the patient arrives in the emergency care unit to enable a faster admission, triage, and treatment process.<br>- Please choose all options relevant for your hospital: (1) not applicable; (2) Not implemented; (3) With emergency services; (4) with partner outpatient doctors; (5) with hospitals; (6) with control centers; (7) Don't know
			Physicians in the hospital have access to medical information (e.g., initial findings, vital signs, ECGs) even before the patient arrives. This way, medical staff can, for instance, prepare themselves for a patient and organize needed resources before the patient arrives.<br>- (1) not applicable; (2) Yes - using the AKTIN protocol; (3) Yes - using alternative technologies/ protocols; (4) No; (5) Don't know
	Org. control & data mgt.	Data management	The hospital integrates externally generated data, e.g., from other service providers, professional associations, cancer registers or health insurance companies, via interoperable interfaces.<br>- (1) Not implemented to (5) Fully implemented; (6) Don't know

Quality indicator	DR dimension	DR sub-dimension	Examples of relevant DR question(s) and answers
Risk-adjusted inpatient mortality ratio of patients hospitalized for outpatient-acquired pneumonia	Clinical processes	Documentation/ diagnosis	The hospital uses text recognition systems to automatically convert, process and evaluate analog documents into digital information (e.g., medical data).<br>- (1) Not implemented to (5) Fully implemented; (6) Don't know
		Decision support	Is there at least one Clinical Decision Support rule in place that is triggered by Physician Documentation?<br>- (1) Not implemented to (5) Fully implemented; (6) Don't know
		Access to inform.	See hip replacement and osteosynthesis surgery after fracture of the femur above
		Order management	An overview of all services requested within the hospital for patients is provided via the HIS.<br>- (1) Not implemented to (5) Fully implemented; (6) Don't know
		Order & medication management	For employees, it is possible to control clinical work processes digitally with the help of workflows and to be automatically informed about treatment steps (status management).<br>- (1) Not implemented to (5) Fully implemented; (6) Don't know
		Device & location independent flexible working	Is information in the HIS available for inquiry outside the organization (i.e., remote access by clinicians)? - (1) Yes, (2) No, (3) Don't know
			The hospital enables clinical staff to access and process clinical information from the HIS or PDMS regardless of the device. - From (1) Not implemented to (5) Fully implemented, (6) Don't know
	Org. control & data	Data management	The hospital uses data analysis and evaluation tools to forecast medical risks for patients. Detected risks are communicated to clinical employees via automated warnings or notices (as decision support) so that they can intervene at an early stage, reduce risks, and optimize care.<br>- (1) Not implemented to (5) Fully implemented; (6) Don't know
Risk-adjusted ratio of inpatient cases with a new bedsore/ decubitus (excl. decubitus/ ulcers of level/ category 1)	Clinical processes	Docum./ diagn.	See other quality indicators
		Decision support	The digital care and treatment documentation includes checklists, reminder aids or signal functions if necessary (mandatory) entries are incorrect or incomplete.<br>- (1) Not implemented to (5) Fully implemented; (6) Don't know
			Nurses have automated clinical decision support capabilities triggered by nursing documentation. Examples include automatic risk detection for falls, decubitus, pain, malnutrition, incontinence, recommendations regarding appropriate nursing measures, medical guidelines, clinical pathways, evidence from nursing research.<br>- (1) Not implemented to (5) Fully implemented; (6) Don't know
		Order mgt.	See other quality indicators
		Order & medication management	For employees, it is possible to control clinical work processes digitally with the help of workflows and to be automatically informed about treatment steps (status management).<br>- (1) Not implemented to (5) Fully implemented; (6) Don't know
	Organizational control & data management	Data management	The hospital uses data analysis and evaluation tools to forecast medical risks for patients. Detected risks are communicated to clinical employees via automated warnings or notices (as decision support) so that they can intervene at an early stage, reduce risks, and optimize care.<br>- (1) Not implemented to (5) Fully implemented; (6) Don't know
			Is the HIS capable of reporting the timeliness of scheduled orders and tasks for physicians, nurses, and other clinical staff?<br>- (1) Yes, this is technically possible, but this feature is currently not used (e.g., due to works council agreements); (2) Yes, this is technically possible, and this feature is used for process optimization; (3) No, this is technically not possible; (4) Don't Know

Annotations: AKTIN = Aktionsbündnis zur Verbesserung der Kommunikations- und Informationstechnologie in der Intensiv- und Notfallmedizin (*Action Alliance for the Improvement of Communication and Information Technology in Intensive and Emergency Medicine*); DICOM = Digital Imaging and Communications in Medicine; Docum./ diagn = Documentation/ diagnosis; DR = DigitalRadar; HIS = Hospital Information System; mgt. = Management; Org. = Organizational; PDMS = Patient Data Management System;. All DR questions can be found in [2].

Sensitivity analysis I – matching observation period of digitalization and quality indicators

Table 8: Sensitivity analysis I – matching observation period of digitalization and quality indicators

	Dependent variable: Value for respective quality indicator	
	(a) Univariate regression analysis	(b) Multivariate regression analysis
**Preop waiting hip replacement (N=665)**		
Model (1): Total DR-score	-0.010 (0.020)	0.007 (0.022)
R^2 (adj.)	-0.001	0.072
Model (2): Sum of sub-dimensions	0.011 (0.367)	0.120 (0.386)
R^2 (adj.)	-0.002	0.072
Model (3): Separate sub-dimensions		
Clinical Processes		
Documentation/ Diagnosis	-2.449 (1.683)	-1.657 (1.699)
Decision support	1.333 (1.369)	1.685 (1.373)
Access to information	0.852 (1.607)	0.422 (1.634)
Telehealth		
Emergency department	-0.662 (1.177)	-0.960 (1.277)
Organizational Control & Data Management		
Data management	0.624 (1.559)	0.438 (1.574)
R^2 (adj.)	-0.003	0.070
**Preop waiting osteosynthesis (N=674)**		
Model (1): Total DR-score	-0.004 (0.021)	-0.001 (0.023)
R^2 (adj.)	-0.001	0.034
Model (2): Sum of sub-dimensions	-0.376 (0.361)	-0.492 (0.395)
R^2 (adj.)	0.000	0.036
Model (3): Separate sub-dimensions		
Clinical Processes		
Documentation/ Diagnosis	-1.563 (1.675)	-1.980 (1.659)
Decision support	0.355 (1.337)	0.776 (1.380)
Access to information	-0.718 (1.447)	-0.609 (1.515)
Telehealth		
Emergency department	-0.977 (1.297)	-1.571 (1.439)
Organizational Control & Data Management		
Data management	0.985 (1.717)	0.464 (1.755)
R^2 (adj.)	-0.004	0.034
**Mortality pneumonia (N=1,126)**		
Model (1): Total DR-score	-0.001 (0.001)	-0.040** (0.017)
R^2 (adj.)	-0.001	0.126
Model (2): Sum of sub-dimensions	-0.007 (0.016)	-0.004*** (0.002)
R^2 (adj.)	-0.001	0.127
Model (3): Separate sub-dimensions		
Clinical Processes		
Document./ Diagn.	-0.121 (0.132)	-0.078 (0.128)
Decision support	-0.034 (0.100)	-0.071 (0.096)
Access to inform.	0.269*** (0.102)	0.159 (0.101)
Order mgt.	0.023 (0.094)	-0.036 (0.091)
Order & med. mgt.	0.091 (0.102)	0.071 (0.101)
Flexible working	-0.134** (0.065)	-0.150** (0.061)
Organizational Control & Data Management		
Data management	-0.143 (0.121)	-0.174 (0.115)
R^2 (adj.)	0.008	0.129

**New decubitus cases (N=1,566)**		
Model (1): Total DR-score	0.014*** (0.002)	0.007*** (0.002)
R^2 (adj.)	0.048	0.141
Model (2): Sum of sub-dimensions	0.196*** (0.023)	0.089*** (0.024)
R^2 (adj.)	0.044	0.140
Model (3): Separate sub-dimensions		
Clinical Processes		
Document./ Diagn.	0.279** (0.140)	0.252* (0.133)
Decision support	0.177 (0.120)	0.124 (0.116)
Order mgt.	0.341*** (0.087)	0.081 (0.087)
Order & med. mgt.	0.062 (0.123)	-0.003 (0.121)
Organizational Control & Data Management		
Data management	0.026 (0.134)	-0.057 (0.128)
R^2 (adj.)	0.045	0.139

Annotations: N = number of observations. Quality data was averaged between 2020 and 2021 to increase variable robustness potentially negatively influenced by the COVID-19 pandemic. In the multivariate regression analysis, we control for federal states, bed categories, ownership, emergency level, teaching hospital status and university hospital type. Coefficients are available from the corresponding author upon request. Asterisks indicate the significance level: *** $p < 0.01$, ** $p < 0.05$, * $p < 0.10$. Numbers in the table indicate beta coefficients, heteroskedasticity-robust standard errors are in parentheses.

Sensitivity analysis II – Inclusion of quality indicator outliers

Table 9: Sensitivity analysis II – Inclusion of quality indicator outliers

	Dependent variable: Value for respective quality indicator	
	(a) Univariate regression analysis	(b) Multivariate regression analysis
**Preop waiting hip replacement (N=591)**		
Model (1): Total DR-score	0.007 (0.028)	-0.003 (0.030)
R^2 (adj.)	-0.002	0.053
Model (2): Sum of sub-dimensions	0.137 (0.463)	-0.005 (0.515)
R^2 (adj.)	-0.002	0.053
Model (3): Separate sub-dimensions		
Clinical Processes		
Documentation/ Diagnosis	-0.853 (2.469)	-0.675 (2.456)
Decision support	-1.454 (1.81)	-0.441 (1.86)
Access to information	1.928 (1.837)	1.098 (1.967)
Telehealth		
Emergency department	0.071 (1.624)	0.076 (1.827)
Organizational Control & Data Management		
Data management	1.438 (2.265)	0.169 (2.265)
R^2 (adj.)	-0.005	0.047
**Preop waiting osteosynthesis (N=533)**		
Model (1): Total DR-score	0.031 (0.036)	0.004 (0.036)
R^2 (adj.)	0.000	0.061
Model (2): Sum of sub-dimensions	0.184 (0.558)	-0.149 (0.557)
R^2 (adj.)	-0.002	0.061
Model (3): Separate sub-dimensions		
Clinical Processes		
Documentation/ Diagnosis	1.031 (2.61)	0.987 (2.479)
Decision support	-2.71 (1.876)	-1.682 (1.878)
Access to information	-0.422 (2.463)	-1.49 (2.632)
Telehealth		
Emergency department	1.032 (1.898)	1.624 (2.051)
Organizational Control & Data Management		
Data management	3.258 (2.639)	0.689 (2.607)
R^2 (adj.)	-0.003	0.056
**Mortality pneumonia (N=1,093)**		
Model (1): Total DR-score	-0.004* (0.002)	-0.004** (0.002)
R^2 (adj.)	0.003	0.038
Model (2): Sum of sub-dimensions	-0.014 (0.019)	-0.013 (0.021)
R^2 (adj.)	0.000	0.034
Model (3): Separate sub-dimensions		
Clinical Processes		
Document./ Diagn.	-0.183 (0.152)	-0.190 (0.159)
Decision support	-0.089 (0.105)	-0.042 (0.102)
Access to inform.	0.348** (0.145)	0.301** (0.135)
Order mgt.	0.041 (0.124)	0.080 (0.120)
Order & med. mgt.	-0.148 (0.111)	-0.221* (0.118)
Flexible working	-0.266*** (0.090)	-0.210** (0.083)
Organizational Control & Data Management		
Data management	0.373** (0.170)	0.341** (0.165)

R^2 (adj.)	0.030	0.058
**New decubitus cases (N= 1,558)**		
Model (1): Total DR-score	0.023*** (0.008)	0.016* (0.008)
R^2 (adj.)	0.001	0.017
Model (2): Sum of sub-dimensions	0.420* (0.217)	0.307 (0.221)
R^2 (adj.)	0.002	0.018
Model (3): Separate sub-dimensions		
Clinical Processes		
Document./ Diagn.	0.024 (0.524)	0.275 (0.377)
Decision support	2.089 (1.798)	1.977 (1.690)
Order mgt.	-0.141 (0.410)	-0.738 (0.795)
Order & med. mgt.	0.534 (0.625)	0.463 (0.799)
Organizational Control & Data Management		
Data management	-0.414 (0.385)	-0.351 (0.301)
R^2 (adj.)	0.002	0.018

Annotations: N = number of observations. In the above analyses, we include quality indicator outliers outside of the 95%-confidence interval of the sample median. In the multivariate regression analysis, we control for federal states, bed categories, ownership, emergency level, teaching hospital status and university hospital type. Coefficients are available from the corresponding author upon request. Asterisks indicate the significance level: *** p < 0.01, ** p < 0.05, * p < 0.10. Numbers in the table indicate beta coefficients, heteroskedasticity-robust standard errors are in parentheses.

Bibliography

1. Amelung V, Angelkorte M, Augurzky B, et al (2022) Zwischenbericht - Ergebnisse der ersten nationalen Reifegradmessung deutscher Krankenhäuser

2. DigitalRadar Krankenhaus Konsortium (2021) Instrument zur Evaluierung des Reifegrads der Krankenhäuser hinsichtlich der Digitalisierung [Measurement tool for the evaluation of hospitals' digital maturity]. St. Gallen

3. Pettit L (2013) Understanding EMRAM and how it can be used by policy-makers, hospital CIOs and their IT teams. World Hosp Health Serv 49:7–9

4. Vogel J, Hollenbach J, Haering A, et al (2024) The association of hospital profitability and digital maturity – An explorative study using data from the German DigitalRadar project. Health Policy (New York) 142:. https://doi.org/10.1016/J.HEALTHPOL.2024.105012

5. Institut für Qualitätssicherung und Transparenz im Gesundheitsweisen (IQTIG) (2023) Öffentliche Berichterstattung von Ergebnissen der externen stationären Qualitätssicherung in den Qualitätsberichten der Krankenhäuser. Empfehlungen des IQTIG zum Berichtsjahr 2022. Berlin

6. Klestil T, Röder C, Stotter C, et al (2018) Impact of timing of surgery in elderly hip fracture patients: a systematic review and meta-analysis. Sci Rep 8:. https://doi.org/10.1038/S41598-018-32098-7

7. Simunovic N, Devereaux PJ, Sprague S, et al (2010) Effect of early surgery after hip fracture on mortality and complications: systematic review and meta-analysis. CMAJ 182:1609–1616. https://doi.org/10.1503/CMAJ.092220

8. NICE (2023) Hip fracture: management - clinical guideline. London

9. Institut für Qualitätssicherung und Transparenz im Gesundheitswesen (IQTIG) (2022) Beschreibung der Qualitätsindikatoren und Kennzahlen nach DeQS-RL. Ambulant erworbene

Pneumonie. Endgültige Rechenregeln für das Erfassungsjahr 2021. 232007_50778 Sterblichkeit im Krankenhaus (inkl. COVID-19 Fälle). Berlin

10. Institut für Qualitätssicherung und Transparenz im Gesundheitswesen (IQTIG) (2022) Beschreibung der Qualitätsindikatoren und Kennzahlen nach DeQS-RL. Dekubitusprophylaxe. Endgültige Rechenregeln für das Erfassungsjahr 2021. 52009: Stationär erworbener Dekubitalulcus (ohne Dekubitalulcera Grad/Kategorie 1). Berlin

11. Salampessy BH, Portrait FRM, van der Hijden E, et al (2021) On the correlation between outcome indicators and the structure and process indicators used to proxy them in public health care reporting. European Journal of Health Economics 22:1239–1251. https://doi.org/10.1007/S10198-021-01333-W/TABLES/3

12. Institut für Qualitätssicherung und Transparenz im Gesundheitsweisen (IQTIG) (2022) Beschreibung der Qualitätsindikatoren und Kennzahlen nach DeQS-RL. Hüftgelenkversorgung: Hüftendoprothesenversorgung. Endgültige Rechenregeln für das Erfassungsjahr 2021. 54003 Präoperative Verweildauer. Berlin

13. Institut für Qualitätssicherung und Transparenz im Gesundheitsweisen (IQTIG) (2022) Beschreibung der Qualitätsindikatoren und Kennzahlen nach DeQS-RL. Hüftgelenkversorgung: Hüftgelenknahe Femurfraktur mit osteosynthetischer Versorgung. Endgültige Rechenregeln für das Erfassungsjahr 2021. 54030 Präoperative Verweildauer. Berlin

14. Atasoy H, Greenwood BN, McCullough JS (2019) The Digitization of Patient Care: A Review of the Effects of Electronic Health Records on Health Care Quality and Utilization. Annu Rev Public Health 40:487–500. https://doi.org/10.1146/annurev-publhealth-040218-044206
